# Expanding dynamics of the virulence-related gene variations in the toxigenic *Vibrio cholerae* serogroup O1

**DOI:** 10.1186/s12864-019-5725-y

**Published:** 2019-05-09

**Authors:** Zhenpeng Li, Bo Pang, Duochun Wang, Jie Li, Jialiang Xu, Yujie Fang, Xin Lu, Biao Kan

**Affiliations:** 1 State Key Laboratory for Infectious Disease Prevention and Control. National Institute for Communicable Disease Control and Prevention, Chinese Center for Disease Control and Prevention, Beijing, 102206 China; 2 Collaborative Innovation Center for Diagnosis and Treatment of Infectious Diseases, Hangzhou, 310003 China; 3 Key Laboratory of Cleaner Production and Integrated Resource Utilization of China National Light Industry, School of Food and Chemical Engeering, Beijing Technology and Business University, Beijing, 100048 China

**Keywords:** *Vibrio cholerae*, Comparative genome, Virulence-related genes, Core genes, Accessory genes, Duplicated genes

## Abstract

**Background:**

Toxigenic *Vibrio cholerae* serogroup O1 is the causative pathogen in the sixth and seventh cholera pandemics. Cholera toxin is the major virulent factor but other virulence and virulence-related factors play certain roles in the pathogenesis and survival in the host. Along with the evolution of the epidemic strains, the virulence-related genes also experience variation, gain and loss, and lead to genetic divergence in different strains.

**Results:**

In this study, we analyzed the virulence-related gene profiles in the toxigenic serogroup O1 strains isolated from 1923 to 2015, the genomes of which were publicly available. The virulence-related genes of the *V. cholerae* O1 strains were annotated based on the Virulence Factors Database (VFDB). An average of 230.1 virulence-related genes per strain were identified; significant differences in the average numbers were found between the classical and El Tor biotypes, and increasing trends in the number of virulence-related genes along with the isolation years were observed in the El Tor biotype strains. A total of 176 homologs of virulence-related genes were found from these strains, of which 25 belonged to the core genes, suggesting their conservative and necessary roles in *V. cholerae* pathogenesis. We described the diversities of the homologs by defining gene sequence type, and illustrated its association with gene duplication; we found that gene duplication clearly increased the complexity of the gene sequence types in the core virulence-related genes. In addition, we provided virulence-related gene profiles whose genetic characteristic depend on the isolation years from the view of gene gain and loss, variation, gene duplication and gene sequence type number.

**Conclusions:**

Our study reveals the comprehensive variation dynamics of the virulence-related genes in toxigenic *V. cholerae* serogroup O1 during epidemics. The increasing trend for the virulence-related genes may suggest the evolutional advantage of strains by gaining virulence-related genes with diverse functional categories.

**Electronic supplementary material:**

The online version of this article (10.1186/s12864-019-5725-y) contains supplementary material, which is available to authorized users.

## Background

*Vibrio cholerae*, a species belonging to the genus Vibrio, is the causative agent of the severe diarrheal disease, cholera. According to its O antigens, *V. cholerae* has been divided into 206 serogroups, in which O1 and O139 are the main serogroups causing cholera epidemics. According to whether they produce the cholera toxin, *V. cholerae* can be classified into two groups: toxigenic strains and non-toxigenic strains. The cholera toxin plays an important role in its pathogenesis. Most cholera pandemics in history had been caused by toxigenic strains. To date, seven cholera pandemics have occurred. The sixth pandemic was caused by the classical biotype of serogroup O1; while the El Tor biotype of serogroup O1 has replaced the classical biotype in the seventh pandemic [1]. The two biotypes are different in their phenotypic and genotypic characteristics, pathogenic potential, infection modes and human survival abilities [2], whereas the strains responsible for both the sixth and seventh pandemics are toxin-producing.

In recent years, genome sequencing and comparative genomics have been commonly used to investigate the evolution and clonal shifts among genetically distinctive *V. cholera*e isolates. Several studies were carried out to elucidate population genetics of *V. cholerae*. It has been found that the pre 7th pandemic isolates and the 6th pandemic clones are related to the 7th pandemic clone, while the 6th pandemic clone is more distantly related, and non-pathogenic isolates have no clonal structure [3]. The substantial differences between the strains of two biotypes (El Tor vs classical biotype) were observed, and the EL Tor strains responsible for the 7th pandemic are highly clonal [4]. A phylogenetic study with a collection of clinical and environmental isolates of *V. cholerae* from Haiti showed that the population size initially increased and then fluctuated over time, and selection analysis suggested diversification likely was driven by positive selection [5]. The genomic origin and evolution of *V.cholera* have also been extensively described by following studies. The strains of 7th pandemic evolved from a nonpathogenic strain in the Middle East, and then underwent rapid diversification [6]. By using 154 whole-genome sequences of globally and temporally representative *V. cholerae* strains, a study characterized that the seventh pandemic has spread from the Bay of Bengal in at least three independent but overlapping waves with a common ancestor in the 1950s, and identified several transcontinental transmission events [7]. Near recently, genomic history of the seventh pandemic of cholera in Africa was illustrated. The study indicated the past epidemics were attributable to a single expanded lineage, and described the periodicity of lineage introduction and the stable routes of cholera spread [8]. Another recent study has given an integrated view of *V. cholerae* in the Americas. The study analyzed the two of the largest cholera epidemics in modern history, and found that intercontinental introductions of seventh pandemic El Tor *V. cholerae* have caused the two epidemics, further consolidating the importance of local lineages [9].

Virulence-related genes refer to genes whose products can help a micropathogen enter a host and colonize, invade, spread, and cause disease [10]. As an important regulatory factor, virulence-related genes play an important role in the pathogenesis of pathogenic bacteria. Virulence-related genes involve many genes whose products are bacterial toxins, cell surface components related to attachment and protection, and hydrolytic enzymes contributing to pathogenicity. Virulence-related genes can be classified into several categories based on the virulence mechanisms, such as adhesion, invasion, intracellular survival mechanisms, extracellular survival mechanisms, nutrient acquisition. In *V. cholerae*, the recognized virulence and virulence-related factors, such as enterotoxin, colonization factors, protein enzymes and chemotaxis, have been widely reported [11, 12]. The capacity for horizontal gene transfer is responsible for spreading common mechanisms of virulence amongst diverse pathogens. A substantial portion of the virulence-related genes are part of the Integrative and Conjugative Elements (ICEs). It has been reported that the *V. cholerae* epidemic is related to the acquisition of specific ICEs [7, 13]. Therefore, the investigation of the virulence-related gene pool coupled with ICEs is necessary.

The virulence-related genes with evolutionary advantage were fixed over a long evolutionary time period, and then the composition of virulence-related genes (here we defined as a virulence-related gene profile) was formed for each strain. A strain may have a specific virulence-related gene profile as demonstrated by the differences in their sequences and copies in different strains. We present this study based on the hypothesis that the strain with a specific virulence-related gene profile has advantage in evolution; the specific virulence gene profile is the fingerprint for the strain’s adaption to evolution. The variation of the specific virulence-related gene profile will reveal the genetic mechanisms of bacterial virulence. In this study, we retrieved the whole genome sequences of the serogroup O1 toxigenic *V. cholerae* strains from 1923 to 2015, in combination with the Virulence Factors Database (VFDB) [14], and performed a comprehensive analysis of virulence-related genes for toxigenic *V. cholerae* O1.

## Methods

### Genomes of the strains

From GenBank, we selected 302 draft and 12 complete genome sequences of *V. cholerae* serogroup O1 strains carrying the cholera toxin genes *ctxAB*. In total, 314 strains were retrieved. The isolation years and biotype were collected from the publicly available literature or the database. The strains used were isolated in the years 1923 to 2015; of all the strains, 20 are tagged as classical biotypes, while 292 are tagged as El Tor biotypes, the remaining two strains were tagged as unknown. There are one and 11 complete genomes for classical and El Tor biotype strains respectively. The detailed Information of the strains selected was listed in Additional file 1.

### The construction of phylogenetic tree

First, we extracted the core genes of all selected strains by using cd-hit and blast+. As a widely used program for clustering and comparing protein or nucleotide sequences, the cd-hit was used to remove highly homologous genes of reference strain (N16961), and then all genes from all selected strains were searched against the non-redundant gene set got by cd-hit; the gene that present just once in all selected strains were deemed to be core genes. Second, the core genes were aligned using clustalw2 [15] and merged. Then, phyML was used to construct the maximum likelihood (ML) tree [16].

### Annotation of the virulence related genes in 314 strain genomes

The pipeline of the virulence gene annotation of *V. cholerae* strain genomes proceeded as follows: genome-wide de novo prediction for all 314 isolates was carried out using the prokaryotic gene prediction software Prodigal [17], and the amino acid sequences of each gene were obtained. To obtain all the potential virulence-related genes of each strain, the VFDB database was downloaded [14], and the protein sequences of each strain were searched against the VFDB using blastp. In this study, we used two different blastp standards to search and obtain the virulence-related gene sets of each strain: a strict blastp standard (identity cutoff 90% with coverage cutoff 80%, E-value cutoff 1e-5) and a relaxed blastp standard (identity cutoff 30% with coverage cutoff 60%, E-value cutoff 1e-5). First, the virulence-related genes in VFDB were screened using the strict blastp standard, and defined as high confidence; then the protein sequences of each strain were searched against the virulence-related genes with high confidence using a relax blast standard; thus, the variant sequence types for the virulence-related genes of high confidence were also included. Third, the blastp results obtained in the second step were screened. Each gene for each strain retained only one potential virulence-related gene with the highest identity. Using these steps, not only did we obtain the virulence-related genes with high confidence, but we also obtained the variant form for these virulence-related genes with high confidence.

### Pan virulence-related genes analysis

The homologous genes may be derived from a common ancestral gene. Ordinarily, these genes were called as homolog by short. As a widely used program for clustering and comparing protein or nucleotide sequences, the cd-hit was used to remove highly homologous genes from potential virulent gene sets [18]. The sequence identity threshold (c) was set as 0.6 and the length difference cutoff (s) was set as 0.8. The protein sequences of the virulence-related genes from VFDB were clustered into hundreds of homologs. Then, the non- redundant sequence set generated by cd-hit was searched against the protein sequences of all 314 strains by blastp to count the number of each homolog group.

### Gene sequence typing

We divided each homolog into various sequence types as follows. First, we obtained all the sequences of each homolog. ClustalW omega was used to perform the multiple alignment [19]. Then, we computed the pair-wise identities among the sequences of each homolog, and the sequence pairs with an identity larger than 0.95 were linked to construct a network for each homolog. Then, the MCL, a sort of Markov Cluster Algorithm, was used to perform the clustering, and inflation was set to 1.4. Finally, the sequences, that were grouped in the same cluster were defined as a gene sequence type, while each sequence that was included in the network as a separated node (not linked with the other nodes) was defined as unique gene sequence type. We have chosen three identity cutoffs (0.975, 0.95, 0.925) to construct the network for each homolog, and got average 10.7, 8.2 and 7.3 gene sequence types, respectively. So we chose 0.95 as cutoff, since the variation of gene sequence types from 0.95 to 0.925 was more stable than that from 0.975 to 0.95.

### KEGG pathway enrichment analysis

To investigate whether a specific gene set is significantly enriched in some KEGG pathways, KOBAS was used to perform the KEGG pathway enrichment analysis [20], N16961 was used as reference strain, and the cutoff of the corrected *p* value was set as 0.05.

### The comparison of global correlation (the number of virulence-related genes vs. the isolation years of the strains) with that of random conditions

To eliminate the impact of disequilibrium of data on the results, we divided the strains into three groups based on the epidemic years of *V. cholerae*, which were before 1961 (before the seventh pandemic), 1961–1992 (early stage of the seventh pandemic) and after 1992 (after the emergence of serogroup O139 and the atypical El Tor biotype). In each round of sampling, we sampled the same number of strains (*n* = 60) with replacement from the three periods, and got 180 strains. Then an actual global correlation coefficient was computed. We also computed a random global correlation coefficient in random conditions when the isolation years were shuffled. Finally, we did 1000 rounds of sampling, and got 1000 actual global correlation coefficients and 1000 random global correlation coefficients. Meanwhile, the Student’s t-Test was used to test the difference between actual global correlation coefficients and that of random conditions.

### Heatmap analysis

First, we constructed a 314 × 176 matrix using 314 strains and 176 homologs. If the strain had a virulence gene, the identity score was filled in at the corresponding position in the matrix. Otherwise, a 0 was filled. The row was arranged by the isolation years of the strains. The pheatmap package in R was used to draw the heatmap. To keep the order of the isolation years, the row was not clustered.

### The correlation analysis between the virulence-related genes and isolation years of strains

As a statistical method, correlation analysis can be used to evaluate the possible connections between variables. To test the correlation between each homolog and the isolation years of the strains, we analyzed their correlation from several perspectives: the gain and loss of virulence-related genes, the variation in the virulence-related genes, the copy number of duplicated genes in each genome and the number of gene sequence types in each genome. First, for each homolog, we check whether existed in each strain or not. If it existed, we compute its distance to the most recent common ancestor (MRCA) of classical biotype (If all the homologous genes belong to El Tor biotype strains, the MRCA of El Tor biotype is used), the number of gene sequence types in that strain and the copy number of the duplicated genes in that strain.

To analyze the correlation between the gain and loss of virulence-related genes and the isolation years of the strains, we first constructed vector A with the same length as the strain number for each homolog, and then, we checked the homolog whether existed in each strain. If the homolog existed in the strain, it is recorded as 1 in A. Otherwise, recorded as 0 in A. Meanwhile, we constructed vector B containing the isolation years of all the strains corresponding to A. Finally, the correlation was analyzed between A and B using the *cor.test* function in R, the *p* value of each correlation was computed, all the *p* values were corrected by False discovery rate (FDR), and the adjusted p value cutoff that we used was 0.01.

To analyze the correlation between the variation in the virulence-related genes and the isolation years of the strains, the computation process was the same as that for the gain and loss of virulence-related genes, except in this process, the values of vector A should be filled with the distance to MRCA. Only the items of A that were not equal to 0 and the corresponding items of B were retained for the correlation analysis.

To analyze the correlation between the copy number of the duplicated genes in each genome and the isolation years of the strains, we selected the homologs with duplicated genes in more than one strain. The computation process was also the same as that for the gain and loss of virulence-related genes, except in this process, the values of vector A should be filled with the copy number of the duplicated genes in that strain of the homolog.

The correlation between the number of gene sequence types in each genome and the isolation years of the strains was the same as that for the copy number of the duplicated genes, except that, in this process, the values of vector A should be filled with the number of gene sequence types in the strain of that homolog.

## Results

### The phylogenetic characterization based on whole genomes

**Fig. 1:**
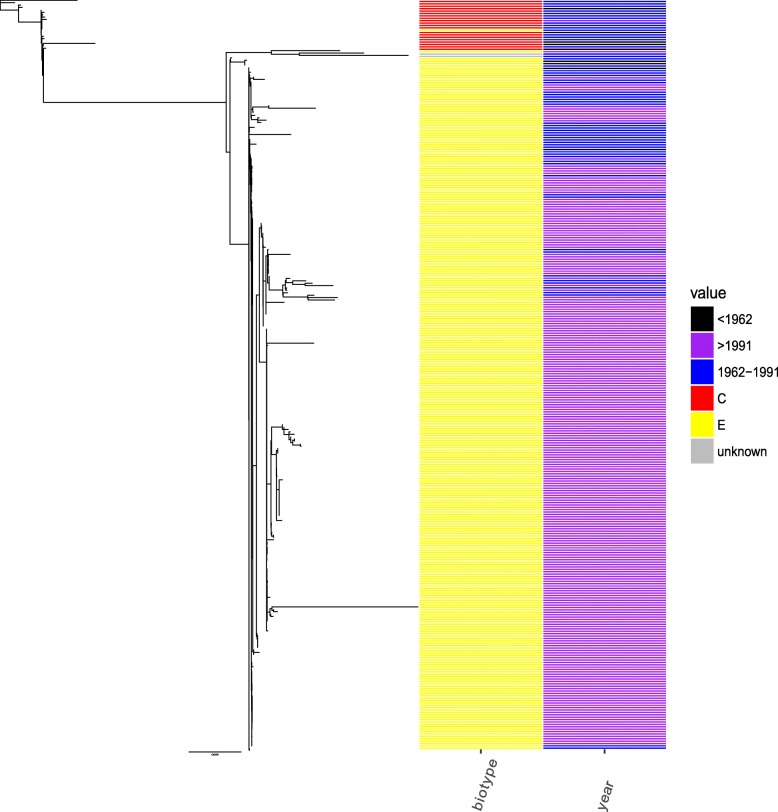
A maximum-likelihood phylogenetic tree for 314 strains of *V. cholerae* based on core genes. The biotype and isolation year for each strain was highlighted in the right

The genome sizes of all selected strains range from 3.8 Mb to 4.8 Mb. The number of coding sequences per genome ranges from 3468 to 4778. The 462 core genes of all selected strains were concatenated to construct the phylogenetic relationships (Fig. 1). The result showed that two biotypes were clearly separated and all strains of classical biotype were clustered together. While the strains of El Tor biotype were clustered in several other clusters. The tree also showed a strong temporal signature, which was consistent with previous results [7]. Strains isolated before 1962 were sporadic distributed among strains isolated between 1962 and 1992. While nearly all strains isolated after 1992 were in the same large cluster.

### The correlation between the number of virulence-related genes and the isolation years of the strains

**Fig. 2:**
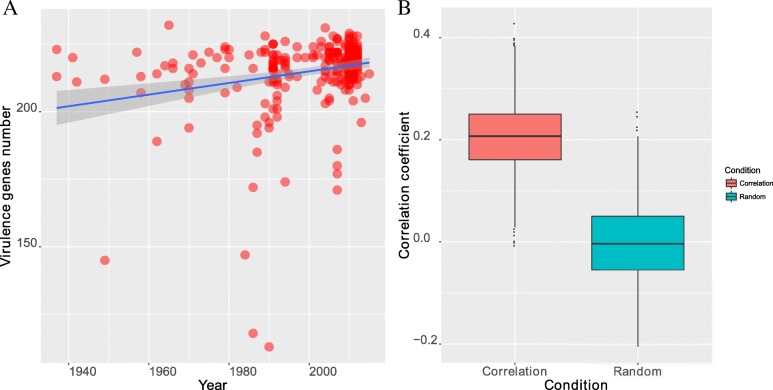
The global relationship between the number of virulence-related genes and the isolation years of the strains. (**a**) A scatter plot between two variables. (**b**) The distribution of actual correlation coefficients and random correlation coefficients between two variables

**Fig. 3:**
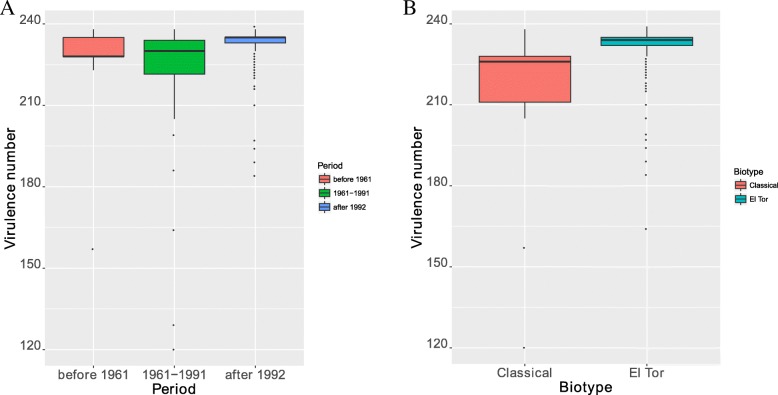
The distribution for the number of virulence-related genes in the different biotypes and epidemic periods of *V. cholerae* serogroup O1. (**a**) The distribution of the number of virulence-related genes in classical and El Tor biotypes. C indicates the classical biotype, E indicated the El Tor biotype. (**b**) The distribution of the number of virulence-related genes in three epidemic periods

We annotated the virulence-related gene profiles of 314 *V. cholerae* O1 strains using blast against the protein sequences of the bacterial virulence gene database VFDB [14]. Each strain has 230.1 virulence-related genes on average. The virulence-related genes in VFDB were collected from various species. We found that the virulence-related genes of *V. cholerae* O1 strains belong to two main species sources: *V. cholerae* and *V. vulnificus,* which account for 94.2 and 5.8%, respectively. The global relationship between the number of virulence-related genes and the years of isolation were displayed in Fig. 2a. A significant linear correlation between the amount of the virulence-related genes of the strains and their isolation years (*p*-value = 4.9e-4, Spearman rank correlation test) were found. To eliminate the impact of disequilibrium of data on the results, we used a resampling method to compare the actual correlation coefficients with random conditions. The result indicated the actual correlation coefficients were significantly larger than random conditions (Fig. 2b, *p*-value< 2.2e-16, Student’s t-Test). In addition, we compared the average numbers of virulence-related genes for the strains in each epidemic group, the average numbers of virulence-related genes for the strains in each group were 222.6, 222.1 and 232.3 per strain, respectively (Fig. 3a). The number of virulence-related genes among the three groups showed that there were significant differences in the number of virulence-related genes between the three groups (*p*-value = 1.4e-07, Kruskal-Wallis rank sum test). The pairwise comparisons among the three groups indicated that there was a significant difference in the average number of virulence-related genes per strain between the second and third group (*p*-value = 3.9e-08, Wilcoxon rank sum test), and no statistically significant differences were found for the other comparisons (*p*-value> 0.05, Wilcoxon rank sum test). Meanwhile, the variance in the virulence-related gene sequences for the three groups showed a decreasing trend. The variances in the average number of virulence-related genes per strain were 25.4, 22.8, and 7.4, suggesting that the average number of virulence-related genes per strain tends to be stable.

The *V. cholerae* seventh pandemic has spread in three overlapping waves [7], here we also compared the average numbers of virulence-related genes for the strains for three waves, there were no significant differences in the number of virulence-related genes among the three waves(*p*-value = 0.14, Kruskal-Wallis rank sum test). The pairwise comparisons among the three waves indicated that there was a significant difference in the average number of virulence-related genes per strain between the wave 1 and wave 3(*p*-value = 0.04, Wilcoxon rank sum test), and no statistically significant differences were found for the other comparisons (*p*-value> 0.05, Wilcoxon rank sum test).

We also compared the two biotypes by the average number of virulence-related genes per strain. On average, 214.9 and 231.7 virulence-related genes per strain were obtained for the classical biotype and El Tor biotype, respectively. The difference between two biotypes was significant (*p*-value < 2.2e-16, Two-sample Kolmogorov-Smirnov test, Fig. 3b). For the El Tor biotype strains, there was a significant linear correlation between the number of virulence-related genes of the strains and their isolation years (*p*-value of 1.9e-05, Spearman rank correlation test), but no linear correlation was found in the classical biotype strains (*p* = 0.4).

### Pan virulence-related gene analysis and gene sequence typing

In this study, we quoted the bacterial core genes (the genes present in all test strains) and the accessory genes (the genes present only in some strains). Pan genome analysis has been one of the necessary methods for comparative genomic analysis in bacteria [21, 22]. The pan virulence-related genes include virulence-related genes that are present in all strains (core virulence-related genes) and virulence-related genes that are only present in some strains (accessory virulence-related genes). Here, we found 176 homologs in which virulence-related genes were present in at least two strains, with 25 genes that were the core virulence-related genes (Table 1). Each homolog covers 302.6 strains on average. There are 142 homologs that appeared only once in each strain, while 34 other homologs were present at least twice in one or more strains, meaning that they were duplicated genes, which have on average 2.8 copies in each strain. The full information of the 176 homologs was provided in Additional file 2.

**Table 1:** The homologs of the virulence-related genes in *V. cholerae* with a coverage ratio for all the strains in the top 20%

Name	Total	Unique	Copy	Genotype	GeneNum/ Genotype	Core	Significance			
							①	②	③	④
*ctxA*	317	314	1	6	52.83	+				–
*ctxB*	320	314	1	10	32	+				
*flrA*	919	314	1	10	91.9	+		+	+	+
*fleR/flrC*	1517	314	1	23	65.96	+				
*vasJ*	314	314	0	6	52.33	+				
*mshJ*	314	314	0	5	62.8	+				+
*mshF*	314	314	0	3	104.67	+				
*rtxB*	3473	314	1	57	60.93	+				
*viuC*	3984	314	1	41	97.17	+		+	+	+
*tlh*	314	314	0	3	104.67	+				
*epsM*	314	314	0	2	157	+				+
*epsG*	314	314	0	1	314	+				
*viuD*	922	314	1	8	115.25	+				
*vctD*	314	314	0	3	104.67	+				
*flaA*	1536	314	1	32	48	+		+	+	
*flgE*	314	314	0	1	314	+				
*flgD*	314	314	0	2	157	+				
*cheV*	1246	314	1	15	83.07	+		+	+	
*fliQ*	314	314	0	1	314	+				
*cheW*	944	314	1	9	104.89	+				+
*cheB*	874	314	1	17	51.41	+		+	+	+
*cheZ*	314	314	0	2	157	+				
*cheY*	1282	314	1	30	42.73	+		–	–	+
*epsB*	314	314	0	4	78.5	+				+
*flgP*	314	314	0	3	104.67	+				+
*acfB*	2059	313	1	32	64.34	–		+	+	
*ace*	316	313	1	1	316	–				+
*vasC*	313	313	0	4	78.25	–				+
*vasD*	313	313	0	2	156.5	–				+
*vasI*	313	313	0	3	104.33	–				+
*vipB/mglB*	313	313	0	2	156.5	–				+
*mshM*	319	313	1	6	53.17	–				–
*mshG*	313	313	0	3	104.33	–				
*mshD*	313	313	0	2	156.5	–				
*rtxB*	1466	313	1	13	112.77	–		+	+	

Note: Total: the number of genes in all strains; Unique: the number of strains having that gene; Copy: if the gene has duplicated genes in more than one strains, it tagged as 1, otherwise as 0. Genotype: the number of genotypes for the homolog. GeneNum/Genotype: the average number of genes of the homolog contributing to a genotype, i.e., the number of genes per genotype. Core: “+” indicates that it is a core virulence gene,“-” indicate it is not a core virulence gene. Significance: “+” indicates it is positive correlated with isolation years of strains, while “-” indicates it is negatively correlated with them; ① indicated the gain and loss of the gene;② indicated the number of genotypes of that homologous gene; ③ indicated the duplicated gene number in each strain; ④ indicated the gene variation

**Fig. 4:**
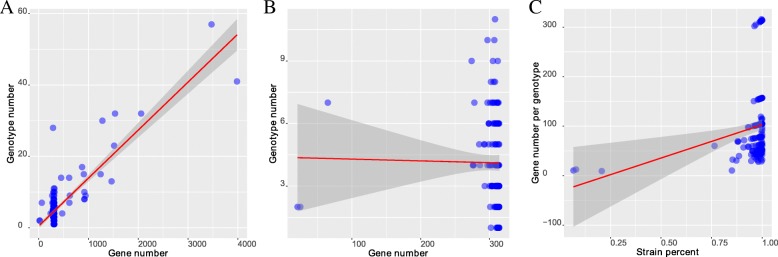
The contribution of duplicated genes to the complexity of the homologous virulence-related genes. (**a**). the correlation between the number of genes in the homolog and the number of gene sequence types in the corresponding group; (**b**). the correlation between the number of genes in the homolog and the number of gene sequence types in the corresponding group when the homolog containing duplicate genes was removed from the analysis; (**c**). the correlation between the average number of genes contributing to a genotype in each homolog and the coverage ratio of the corresponding homolog to all strains

To investigate the extent of variation within each homolog, we subtyped each homolog based on its sequence variations. Here, we defined the gene sequence type (see methods for details). The gene sequence types range from 1 to 57 with an average of 6.0, and the average gene sequence types of the core virulence-related genes and accessory virulence-related genes were 11.8 and 5.0, respectively. The average numbers of genes contributing to a genotype for the core virulence-related genes and accessory virulence-related genes were 114.9 and 95.1, respectively. Furthermore, we tested the correlation between the number of genes in the homolog and the number of gene sequence types in the corresponding group. We found a significant correlation between them (*p*-value < 2.2e-16, Pearson’s product moment correlation coefficient is 0.8, Fig. 4a). In other words, the number of gene sequence types in the homolog increased with gene number, while the significant correlation between them disappeared if we removed homologs containing duplicate genes (*p*-value = 8.5e-1, Pearson’s product moment correlation coefficient is − 1.6e-2, Fig. 4b). This suggests that duplicated genes are important factors contributing to this association. If we remove the core homologs containing duplicate genes, the average number of genes contributing to a genotype for the core virulence-related genes increased to 155.8, which was 35.6% higher than the situation when core homologs containing duplicate genes not removed. These results suggest that gene duplication obviously increased the gene sequence types of core virulence-related genes. Furthermore, we tested the correlation between the average numbers of genes contributing to a genotype in each homolog and the coverage ratios of the corresponding homolog to all strains. We found that there was a significant correlation between them (*p*-value = 3.2e-3, correlation coefficient is 2.2e-1, Fig. 4c). This result hints that the higher the coverage ratio of the homolog in the strains, the higher the overall tendency of the homolog to maintain fewer genotypes. Considering the above results, there may be a balance for core virulence-related genes between the diversity driving by gene duplication and the maintenance of functional stability.

### Classification and functional analysis of virulence-related genes associated with isolation years of the strains

**Fig. 5:**
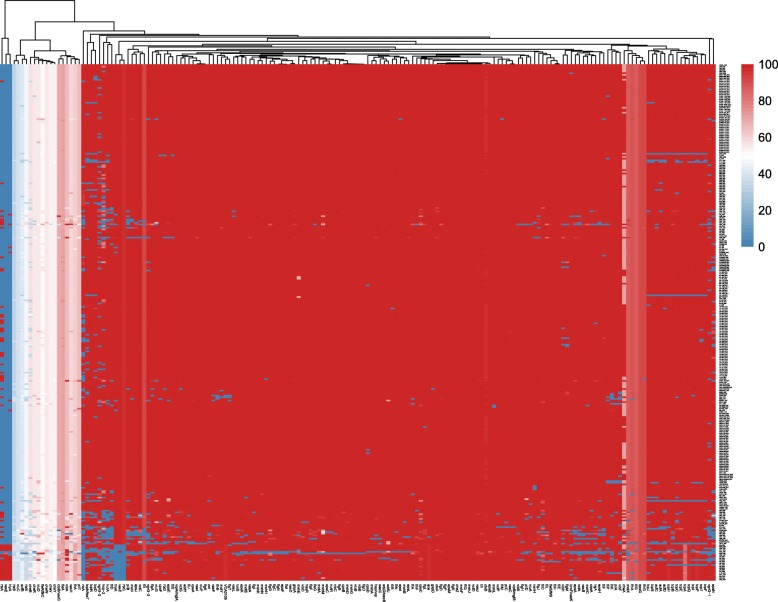
The heatmap representing the relationship between pan virulence-related genes and the isolation years of the strains

During the long-term evolutionary process, virulence-related genes may be gained, lost or experience variation. First, we clustered all the virulence-related genes according to whether the virulence-related genes are present in the genome; as shown in Fig. 5, the virulence-related genes were divided into two large groups. One of the large groups has only three genes, *rtxA*^O395^, *vasE* and *hlyA*, and the appearance of these three virulence-related genes decreased with the isolation years. There are two homologs for *rtxA*, and the difference in their nucleotides is more than 40%. *RtxA* in this group is similar to strain O395. Therefore, we denoted *rtxA* in this group as *rtxA*^O395^. Another larger group can be divided into two subgroups: the first subgroup contained virulence-related gene variations with isolation years, and the virulence-related genes of the second subgroup increased with the number of isolation years.

Next, we made a more precise analysis of the virulence genes, whose genetic characteristics were dependent on the isolation years. First, we screened the virulence-related genes that correlated with isolation years from two perspectives: virulence gene gain and loss and virulence gene variation. From the perspective of gain and loss, we found 30 groups whose homologous virulence-related genes have significantly positive linear correlations with isolation years. KEGG enrichment analysis indicated the pathway enrichment in *V. cholerae* infection, the bacterial secretion system, flagellar assembly and the *V. cholerae* pathogenic cycle. Those that had a significantly negative linear correlation with isolation years were *vasE*, *rtxA* and *hlyA*. Regarding variation, we found 59 groups whose homologous virulence-related genes have a significantly positive linear correlation with isolation years, KEGG pathway enrichment in *V. cholerae* infection, the *V. cholerae* pathogenic cycle, flagellar assembly, bacterial chemotaxis and biosynthesis of the siderophore group nonribosomal peptides, while the number of those with a significant negative linear correlation with isolation years was 14. There was KEGG pathway enrichment in the flagellar assembly and *V. cholerae* infection.

As the copy numbers of duplicated virulence-related genes may vary with the isolation years, we next screened the virulence-related genes that correlated with isolation years from the perspective of copy number. We found 11 groups of homologous virulence-related genes that have significantly positive linear correlation with isolation years and KEGG pathway enrichment in two-component systems. In contrast, those with significantly negative linear correlations with isolation years were *cheA* and *cheY*. Furthermore, we screened the virulence-related genes correlating with isolation years with regard to the gene sequence type number. Six positively correlated genes, whose KEGG pathway enrichment in two-component systems and bacterial chemotaxis, were found. One negatively correlated gene, *cheY*, was also found. The detailed information for the homologs with a top 20% coverage ratio is listed in Table 1. The detailed results of KEGG enrichment analysis for virulence-related gene sets correlating with isolation years from different perspectives were listed in Additional file 3.

Regarding virulence gene gain and loss, copy number and gene sequence type number, we found that the number of virulence-related genes with a significant positive correlation was clearly larger than of the number of virulence-related genes with a negative correlation. These results indicated that some virulence-related genes tend to cover more strains, have higher copy numbers and more gene sequence types.

## Discussion

Virulence-related genes play an important role in the pathogenesis of bacteria. It is well known that cholera toxin plays a key role in *V. cholerae* infection,the regulation of virulence-related genes in *V. cholerae* is complex and linked very closely to other regulatory pathways in the cell [23, 24]. Update, due to the high cost of complete genome sequencing, draft genomes have been widely accepted and used in comparative genomic studies, such as pan-genome analysis, virulence and resistance. In this study, we analyzed the genomic changes of virulence-related genes with isolation years in *V. cholerae* toxigenic strains for the O1 serogroup from 1923 to 2005.

We annotated the virulence-related genes for 314 strains of *V. cholerae*, and found that there was a significant linear correlation between the numbers of virulence-related genes of each strain and their isolation years. However, if we tested the correlation above using the El Tor or classical biotype separately, we found that there was no that type of significant correlation. These results indicated that the global correlation was a result of the El Tor biotypes, but we cannot rule out that the lack of a significant correlation for the classical biotype was caused by the small sample size. We also found that there were significant differences in the number of virulence-related genes between the strains of three pandemic periods. The average number of virulence-related genes for each strain in the third group (the strains after 1992) is much higher than that of the second groups. In previous studies, it has been found that the seventh pandemic of *V. cholerae* spread from the Bay of Bengal in at least three independent, but overlapping waves, and the transmission was coupled with the acquisition of Integrative and Conjugative Elements (ICEs), such as VSP-2 and SXT [7, 13, 25]. Our results further confirmed the fixation of virulence-related genes in the *V. cholerae* genome during the seventh pandemic.

Pan virulence-related gene analysis may help to identify virulence gene pools and understand the diversity of virulence-related genes in toxigenic *V. cholerae* O1 strains. The pan virulence gene analysis identified 25 core homologous virulence-related genes. The function of these genes involves chemotaxis, secretion systems, toxins*,* etc. This suggests that the role of these virulence-related genes in the pathogenicity and epidemic dispersal of *V. cholerae*. These genes should be inherent in *V. cholerae*, or in the early stages of strain evolution. In addition, considering their high conservation, these genes can also be used as targets for the detection of *V. cholerae*. The other non-core virulence genes may be acquired by horizontal gene transfer. These genes may confer an evolutionary selection advantage in certain environmental conditions for the strains [22].

As a fast and scalable unsupervised cluster algorithm for graphs [26], the MCL algorithm has been widely used in the field of gene family and ortholog group detection [27, 28]. Here, the genotype number and number of contributing genes per genotype derived from MCL algorithm were used as intuitive measures for the diversity of homolog. Gene duplication is considered to be a driving force for creating new genes, which could lead to a higher gene dosage, has a key role in gene regulatory network evolution, and may be associated with environmental adaptation of the prokaryote [29–31]. By comparing the genotype information between the homologs with the duplicated genes and those without duplicated genes, our findings indicate that the duplication of virulence-related genes play key part in driving the diversity of core virulence-related genes in *V. cholerae*.

We screened several virulence-related gene sets that were correlated with isolation years from various perspectives: virulence gene gain and loss, copy number and gene sequence type number. The virulence-related genes that were simultaneously screened in several conditions may reflect the evolutionary trend in *V. cholerae*, which may result from the positive selection driven by the pressure of survival and population size increasing.

## Conclusions

Our study characterized the global variation dynamics of virulence-related genes in toxigenic *V. cholerae* serogroup O1. The increasing trend for the virulence-related genes indicates that we should pay more attention to those virulence genes that recently emerged or show recent genetic variation. The understanding of the core virulence genes obtained by the pan virulence-related gene analysis gave us clues about the fundamental virulence mechanism. As gene duplication obviously increases the complexity of the gene sequence types in the core virulence-related genes, we should pay more attention to the roles gene duplication played in virulence evolution. The results will provide key information for *V. cholerae* with respect to virulence-related gene evolution and virulence surveillance, indicating that strains with more virulence-related genes may have an evolutionary advantage.

## Additional files

Additional file 1: **Table S1.** The detailed Information of the strains selected in this study. (XLS 90 kb)

Additional file 2: **Table S2.** The detailed information of 176 virulence-related homologs in which each homolog was present in at least two strains. (XLS 65 kb)

Additional file 3: **Table S3**. The detailed results of KEGG enrichment analysis for virulence-related gene sets correlating with isolation years from different perspectives. (XLS 40 kb)

## Abbreviations

FDR: False discovery rate
ICE: Integrative and Conjugative Elements
ML: Maximum likelihood
MRCA: Most recent common ancestor
VFDB: Virulence Factors Database
